# *N*,*N*′-(1,4-Phenyl­ene)bis­(4-chloro­butanamide)

**DOI:** 10.1107/S1600536812001341

**Published:** 2012-01-18

**Authors:** Olesea Cuzan, Sergiu Shova, Constantin Turta, Ionel I. Mangalagiu

**Affiliations:** a Institute of Chemistry of the Academy of Sciences of Moldova, 3 Academiei Street, Chisinau MD-2028, Moldova; b Institute of Applied Physics of the Academy of Science of Moldova, 5 Academiei Street, Chisinau MD-2028, Moldova; c Institute of Macromolecular Chemistry "Petru Poni", 41A Grigore Ghica Voda Alley, Iasi-700487, Romania; d "Alexandru Ioan Cuza" University, Organic Chemistry Department, 11 Carol I Boulevard, Iasi-700506, Romania

## Abstract

The title mol­ecule, C_14_H_18_Cl_2_N_2_O_2_, lies on a crystallographic inversion center and the each 4-chloro­butanamide group adopts an *anti*-staggered conformation. In the crystal, adjacent mol­ecules are linked through N—H⋯O contacts, forming infinite ribbons extending parallel to the *a* axis.

## Related literature

For details and syntheses of chloro­amides as precursors for new aza­macrocycles see: Benaglia *et al.* (2005 ▶); Harte & Gunnlaugsson (2006 ▶); Humphrey & Chamberlin (1997 ▶); Mangalagiu *et al.* (2007 ▶); Zbancioc *et al.* (2012 ▶).
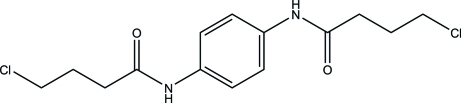


## Experimental

### 

#### Crystal data

C_14_H_18_Cl_2_N_2_O_2_
*M* *_r_* = 317.20
Triclinic, 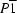

*a* = 5.105 (5) Å
*b* = 6.876 (5) Å
*c* = 10.549 (5) Å
α = 97.735 (5)°
β = 93.214 (5)°
γ = 90.512 (5)°
*V* = 366.3 (5) Å^3^
*Z* = 1
Mo *K*α radiation
μ = 0.45 mm^−1^
*T* = 200 K
0.25 × 0.2 × 0.2 mm

#### Data collection

Agilent Xcalibur Eos diffractometer
Absorption correction: multi-scan (*CrysAlis PRO*; Agilent, 2011 ▶) *T*_min_ = 0.914, *T*_max_ = 1.000
2575 measured reflections
1446 independent reflections
1189 reflections with *I* > 2σ(*I*)
*R*_int_ = 0.026

#### Refinement

*R*[*F* ^2^ > 2σ(*F* ^2^)] = 0.038
*wR*(*F* ^2^) = 0.092
*S* = 1.03
1446 reflections
91 parameters
H-atom parameters constrained
Δρ_max_ = 0.23 e Å^−3^
Δρ_min_ = −0.26 e Å^−3^

### 

Data collection: *CrysAlis PRO* (Agilent, 2011 ▶); cell refinement: *CrysAlis PRO*; data reduction: *CrysAlis PRO*; program(s) used to solve structure: *SHELXS97* (Sheldrick, 2008 ▶); program(s) used to refine structure: *SHELXL97* (Sheldrick, 2008 ▶); molecular graphics: *ORTEP-3* (Farrugia, 1997 ▶); software used to prepare material for publication: *SHELXL97*.

## Supplementary Material

Crystal structure: contains datablock(s) I, global. DOI: 10.1107/S1600536812001341/nk2130sup1.cif

Structure factors: contains datablock(s) I. DOI: 10.1107/S1600536812001341/nk2130Isup2.hkl

Supplementary material file. DOI: 10.1107/S1600536812001341/nk2130Isup3.cml

Additional supplementary materials:  crystallographic information; 3D view; checkCIF report

## Figures and Tables

**Table 1:** Hydrogen-bond geometry (Å, °)

*D*—H⋯*A*	*D*—H	H⋯*A*	*D*⋯*A*	*D*—H⋯*A*
N1—H1⋯O1^i^	0.88	2.10	2.941 (3)	161

Symmetry code: (i) 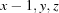.

**Fig. 1.**
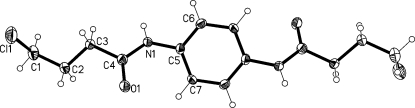
The molecular structure of C14H18Cl2N2O2. Displacement ellipsoids are drawn at 50% probability level. H atoms are presented as small spheres of arbitrary radius. Symmetry code: (i) –x, –y+1, –z+1.

**Fig. 2.**
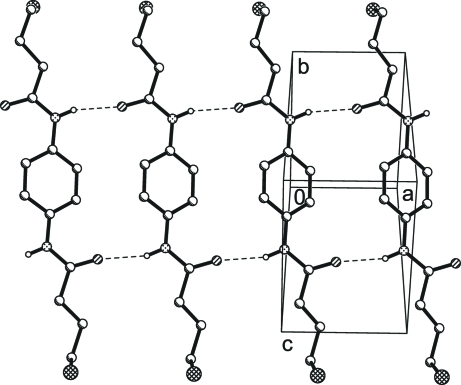
Part of the crystal structure of C14H18Cl2N2O2. Molecular chains generated by N—H···O hydrogen bonds are shown by dashed lines. H atoms not involved in intermolecular bonding have been omitted.

## supplementary crystallographic information

### Comment

With the aim of synthesizing new chloroamides as precursors for new azamacrocycles (Zbancioc *et al.*, 2012), we report the synthesis and crystal structure of the title compound C_14_H_18_Cl_2_N_2_O_2_, which represents a diamide with aliphatic arms, consisting of two moieties of butyryl chloride and a phenylenediamine unit. Amides are important building blocks in preparative macrocycle chemistry (Harte & Gunnlaugsson, 2006), due to their spectroscopic proprieties as well as to their arms ability to coordinate to metal centers. The X-ray structure of the title compound with the atom numbering scheme is shown in Fig. 1. The molecule is assembled from two centro-symmetrically related units through the C_i_ at the center of the aromatic ring. The amide group is rotated by 32.4 (2)° in respect with the phenyl ring. The butyryl chloride fragment adopts an anti-staggered conformation. The main crystal structure motif arises from the parallel packing of the ribbon (Fig. 2) along the crystallographic *a* axis. The infinite ribbons are stabilized *via* intermolecular N1—H1···O1^ii^ H-bond with N1—H1 = 0.88 Å, N1···O1^ii^ = 2.941 (3) Å, [symmetry code ii: *x*–1, *y*, *z*], H1···O1^ii^ = 2.10 Å and N1HO1 angle of 161°.

### Experimental

*p*-Phenylenediamine (5 mmol, 0.54 g) was dissolved in sodium hydroxide solution (0.4 N, 50 ml) and 4-chlorobutyryl chloride (30 mmol, 3.4 ml) was added dropwise under stirring at 0° C for 1 h. Afterwards the mixture was stirred at room temperature overnight resulting in a white precipitate, which was separated by filtration, washed several times with water and dried in vacuum; yield 60%. The purity of *N*,*N*'-(1,4-phenylene)bis(4-chlorobutanamide) was confirmed by ^1^H and ^13^C NMR spectra.

^1^H NMR (DMSO-d_6_) δ (p.p.m.): 9.876 (s, 2NH), 7.492 (s, 4H, Ar), 3.675–3.708 (t, J = 6.8 Hz, 4H, CH_2_, adjacent to chlor), 2.433–2.469 (t, J = 7.2 Hz, 4H, CH_2_, adjacent to amido), 1.988–2.057 (c, J = 6.8 Hz J = 7.2 Hz, 4H, CH_2_).

^13^C NMR (DMSO-d_6_) δ (p.p.m.): 169.72 (2 C, C═O), 134.46 (2 C, Ar), 119.37 (4 C, Ar), 44.97 (2 C, CH_2_, adjacent to chlor), 33.19 (2 C, CH_2_, adjacent to amido), 27.90 (2 C, CH_2_).

### Refinement

The H atoms were positioned geometrically and refined using a riding model with C—H = 0.95–0.99 Å and with *U*_iso_(H) = 1.2 times *U*_eq_(C).

### Crystal data

**Table:**

C_14_H_18_Cl_2_N_2_O_2_	*Z* = 1
*M**_r_* = 317.20	*F*(000) = 166
Triclinic, *P*1	*D*_x_ = 1.438 Mg m^−^^3^
Hall symbol: -P 1	Mo *K*α radiation, λ = 0.71073 Å
*a* = 5.105 (5) Å	Cell parameters from 1244 reflections
*b* = 6.876 (5) Å	θ = 3.0–29.4°
*c* = 10.549 (5) Å	µ = 0.45 mm^−^^1^
α = 97.735 (5)°	*T* = 200 K
β = 93.214 (5)°	Prism, clear light yellow
γ = 90.512 (5)°	0.25 × 0.2 × 0.2 mm
*V* = 366.3 (5) Å^3^	

### Data collection

**Table:**

Agilent Xcalibur Eos diffractometer	1446 independent reflections
Radiation source: fine-focus sealed tube	1189 reflections with *I* > 2σ(*I*)
graphite	*R*_int_ = 0.026
Detector resolution: 16.1593 pixels mm^-1^	θ_max_ = 26.0°, θ_min_ = 3.0°
ω scans	*h* = −5→6
Absorption correction: multi-scan (CrysAlis PRO; Agilent, 2011)	*k* = −8→7
*T*_min_ = 0.914, *T*_max_ = 1.000	*l* = −12→8
2575 measured reflections	

### Refinement

**Table:**

Refinement on *F*^2^	Primary atom site location: structure-invariant direct methods
Least-squares matrix: full	Secondary atom site location: difference Fourier map
*R*[*F*^2^ > 2σ(*F*^2^)] = 0.038	Hydrogen site location: inferred from neighbouring sites
*wR*(*F*^2^) = 0.092	H-atom parameters constrained
*S* = 1.03	*w* = 1/[σ^2^(*F*_o_^2^) + (0.0387*P*)^2^ + 0.0691*P*] where *P* = (*F*_o_^2^ + 2*F*_c_^2^)/3
1446 reflections	(Δ/σ)_max_ = 0.001
91 parameters	Δρ_max_ = 0.23 e Å^−^^3^
0 restraints	Δρ_min_ = −0.26 e Å^−^^3^

### Special details

**Table:**

Geometry. All e.s.d.'s (except the e.s.d. in the dihedral angle between two l.s. planes) are estimated using the full covariance matrix. The cell e.s.d.'s are taken into account individually in the estimation of e.s.d.'s in distances, angles and torsion angles; correlations between e.s.d.'s in cell parameters are only used when they are defined by crystal symmetry. An approximate (isotropic) treatment of cell e.s.d.'s is used for estimating e.s.d.'s involving l.s. planes.
Refinement. Refinement of *F*^2^ against ALL reflections. The weighted *R*-factor *wR* and goodness of fit *S* are based on *F*^2^, conventional *R*-factors *R* are based on *F*, with *F* set to zero for negative *F*^2^. The threshold expression of *F*^2^ > σ(*F*^2^) is used only for calculating *R*-factors(gt) *etc*. and is not relevant to the choice of reflections for refinement. *R*-factors based on *F*^2^ are statistically about twice as large as those based on *F*, and *R*- factors based on ALL data will be even larger.

### Fractional atomic coordinates and isotropic or equivalent isotropic displacement parameters (Å^2^)

**Table:**

	*x*	*y*	*z*	*U*_iso_*/*U*_eq_	
Cl1	0.29972 (11)	−0.27268 (9)	1.02884 (5)	0.0434 (2)	
O1	0.4276 (2)	0.1286 (2)	0.67205 (14)	0.0337 (4)	
C5	0.0022 (3)	0.3420 (3)	0.57107 (17)	0.0189 (4)	
C6	−0.1885 (3)	0.4863 (3)	0.58668 (18)	0.0201 (4)	
H6	−0.3186	0.4768	0.6466	0.024*	
N1	−0.0077 (3)	0.1862 (2)	0.64528 (14)	0.0207 (4)	
H1	−0.1645	0.1441	0.6607	0.025*	
C2	0.3081 (3)	−0.2263 (3)	0.77558 (18)	0.0241 (4)	
H2A	0.2956	−0.2923	0.6860	0.029*	
H2B	0.4926	−0.1826	0.7966	0.029*	
C1	0.2360 (4)	−0.3719 (3)	0.86319 (19)	0.0309 (5)	
H1A	0.3385	−0.4927	0.8437	0.037*	
H1B	0.0476	−0.4077	0.8477	0.037*	
C4	0.2010 (3)	0.0948 (3)	0.69526 (18)	0.0206 (4)	
C3	0.1331 (3)	−0.0471 (3)	0.78585 (18)	0.0229 (4)	
H3A	−0.0520	−0.0909	0.7674	0.027*	
H3B	0.1498	0.0214	0.8748	0.027*	
C7	0.1922 (3)	0.3575 (3)	0.48302 (17)	0.0199 (4)	
H7	0.3238	0.2610	0.4710	0.024*	

### Atomic displacement parameters (Å^2^)

**Table:**

	*U*^11^	*U*^22^	*U*^33^	*U*^12^	*U*^13^	*U*^23^
Cl1	0.0626 (4)	0.0419 (3)	0.0274 (3)	0.0172 (3)	0.0011 (3)	0.0104 (2)
O1	0.0155 (7)	0.0434 (9)	0.0481 (9)	0.0006 (6)	0.0023 (6)	0.0278 (7)
C5	0.0161 (8)	0.0198 (9)	0.0210 (10)	−0.0022 (7)	−0.0029 (7)	0.0060 (8)
C6	0.0154 (8)	0.0251 (10)	0.0203 (9)	−0.0005 (7)	0.0031 (7)	0.0045 (8)
N1	0.0148 (7)	0.0222 (8)	0.0269 (9)	−0.0006 (6)	0.0014 (6)	0.0102 (7)
C2	0.0237 (9)	0.0244 (10)	0.0254 (10)	0.0034 (8)	0.0017 (8)	0.0073 (8)
C1	0.0379 (11)	0.0248 (11)	0.0307 (12)	0.0049 (8)	−0.0020 (9)	0.0075 (9)
C4	0.0173 (9)	0.0206 (10)	0.0243 (10)	0.0012 (7)	−0.0004 (7)	0.0046 (8)
C3	0.0184 (8)	0.0252 (10)	0.0272 (10)	0.0042 (7)	0.0042 (8)	0.0104 (8)
C7	0.0158 (8)	0.0212 (9)	0.0230 (10)	0.0023 (7)	0.0000 (7)	0.0047 (8)

### Geometric parameters (Å, °)

**Table:**

Cl1—C1	1.799 (2)	C2—C3	1.524 (3)
O1—C4	1.222 (2)	C2—H2A	0.9900
C5—C7	1.393 (2)	C2—H2B	0.9900
C5—C6	1.397 (3)	C1—H1A	0.9900
C5—N1	1.412 (2)	C1—H1B	0.9900
C6—C7^i^	1.381 (3)	C4—C3	1.505 (3)
C6—H6	0.9500	C3—H3A	0.9900
N1—C4	1.359 (2)	C3—H3B	0.9900
N1—H1	0.8800	C7—C6^i^	1.381 (3)
C2—C1	1.508 (3)	C7—H7	0.9500
			
C7—C5—C6	118.79 (17)	Cl1—C1—H1A	109.4
C7—C5—N1	123.01 (16)	C2—C1—H1B	109.4
C6—C5—N1	118.20 (16)	Cl1—C1—H1B	109.4
C7^i^—C6—C5	121.48 (17)	H1A—C1—H1B	108.0
C7^i^—C6—H6	119.3	O1—C4—N1	122.99 (17)
C5—C6—H6	119.3	O1—C4—C3	122.17 (16)
C4—N1—C5	126.45 (15)	N1—C4—C3	114.81 (15)
C4—N1—H1	116.8	C4—C3—C2	112.75 (15)
C5—N1—H1	116.8	C4—C3—H3A	109.0
C1—C2—C3	113.03 (16)	C2—C3—H3A	109.0
C1—C2—H2A	109.0	C4—C3—H3B	109.0
C3—C2—H2A	109.0	C2—C3—H3B	109.0
C1—C2—H2B	109.0	H3A—C3—H3B	107.8
C3—C2—H2B	109.0	C6^i^—C7—C5	119.73 (17)
H2A—C2—H2B	107.8	C6^i^—C7—H7	120.1
C2—C1—Cl1	111.34 (14)	C5—C7—H7	120.1
C2—C1—H1A	109.4		
			
C7—C5—C6—C7^i^	0.1 (3)	C5—N1—C4—C3	171.72 (16)
N1—C5—C6—C7^i^	−179.67 (15)	O1—C4—C3—C2	−37.8 (2)
C7—C5—N1—C4	35.9 (3)	N1—C4—C3—C2	144.32 (17)
C6—C5—N1—C4	−144.38 (18)	C1—C2—C3—C4	−178.27 (15)
C3—C2—C1—Cl1	−67.01 (19)	C6—C5—C7—C6^i^	−0.1 (3)
C5—N1—C4—O1	−6.1 (3)	N1—C5—C7—C6^i^	179.66 (16)

Symmetry codes: (i) −*x*, −*y*+1, −*z*+1.

### Hydrogen-bond geometry (Å, °)

**Table:**

*D*—H···*A*	*D*—H	H···*A*	*D*···*A*	*D*—H···*A*
N1—H1···O1^ii^	0.88	2.10	2.941 (3)	161

Symmetry codes: (ii) *x*−1, *y*, *z*.
